# Thermal performance of early stages of *Sparus aurata* integrating body condition, behavior and physiological responses

**DOI:** 10.1038/s41598-025-22781-x

**Published:** 2025-11-06

**Authors:** João Carlos Almeida, Ana Beatriz Costa, Buzenur Ozkan, Sara Martins-Cardoso, Ana Luísa Maulvault, Pedro Pousão-Ferreira, Laura Ribeiro, André Ricardo Araújo Lima, Ana Margarida Faria, Ana Rita Lopes

**Affiliations:** 1 MARE - Marine and Environmental Sciences Centre/ARNET-Aquatic Research Network, ISPA-Instituto Universitário, Lisbon, 1149-041 Portugal; 2 MARE - Marine and Environmental Sciences Centre/ARNET-Aquatic Research Network, Faculty of Sciences, University of Lisbon, Campo Grande, Lisbon, 1749-016 Portugal; 3 IPMA, I.P., Portuguese Institute for the Sea and Atmosphere, I.P., Division of Aquaculture, Upgrading and Bioprospection, Av. Doutor Alfredo Magalhães Ramalho 6, Lisbon, 1495-165 Portugal; 4 UCIBIO — Applied Molecular Biosciences Unit, NOVA School of Science and Technology, NOVA University of Lisbon, Campus de Caparica, Caparica, 2829-516 Portugal; 5 Associate Laboratory i4HB Institute for Health and Bioeconomy, NOVA School of Science and Technology, NOVA University of Lisbon, Caparica, 2829-516 Portugal; 6 Faculdade de Ciências e Tecnologia, Universidade do Algarve, Campus de Gambelas, Faro, 8005-139 Portugal; 7 CIBIO, Centro de Investigação em Biodiversidade e Recursos Genéticos, InBIO Laboratório Associado, Universidade do Porto, Vairão, Portugal; 8 BIOPOLIS Program in Genomics, Biodiversity and Land Planning, CIBIO, Vairão, Portugal; 9 Atlantic International Research Centre, Lisbon, Portugal; 10 Department of Animal Biology, Faculty of Sciences, University of Lisbon, Campo Grande, Lisbon, 1749-016 Portugal

**Keywords:** Agonistic interactions, Marine fish, Metabolism, Oxidative stress, Thermal stress, Physiology, Zoology

## Abstract

Temperature has a profound impact on fish, particularly during the early life stages (ELS), when they exhibit a narrower range of thermal tolerance compared to later developmental phases. This study investigated the long-term effects of temperature on *Sparus aurata* ELS that were exposed to four temperature regimes for 11 weeks: 19 °C, 22 °C, 24 °C and 28 °C. Parameters evaluated included growth, Fulton’s condition factor, behavior (risk-taking, activity and aggressiveness), routine metabolic rate (RMR), metabolic enzyme activity (citrate synthase and lactate dehydrogenase) and oxidative stress biomarkers (Lipid Peroxidation, Catalase and Glutathione-S-Transferase). Growth and condition were highest at 28 °C and lowest at 19 °C. While risk-taking behavior was unaffected by temperature, aggressiveness increased at 24 °C and 28 °C. Swimming activity increased from 22 °C to 28 °C, but RMR remained stable. Antioxidant enzyme activity increased in the brain and gills at 28 °C, but no signs of Lipid Peroxidation were found. Physiological biomarkers accounted for over 67% of trait variability. These findings suggest that S. aurata ELS adopt temperature-specific physiological strategies, such as upregulating metabolic enzymes at lower temperatures to sustain body condition and enhancing antioxidant defenses at higher temperatures to counteract oxidative stress. Thus, ELS can maintain physiological performance across a broad thermal range, albeit with acclimatize trade-offs.

**Supplementary Information:**

The online version contains supplementary material available at 10.1038/s41598-025-22781-x.

## Introduction

Marine habitats are increasingly impacted by rising temperatures, both on daily and seasonal scales, as well as by the growing frequency, duration and intensity of extreme weather events such as marine heatwaves^1–4^. Ectothermic organisms, such as fish, are particularly sensitive to these environmental changes since their body temperature is directly influenced by the surrounding conditions^5^. As the temperature rises, fish must find strategies to meet their physiological requirements, often through trade-offs that impact behavior and survival strategies^6^. Such mechanisms are difficult to assess, as species adopt distinct and contrasting strategies to cope with environmental stressors. This may involve metabolic investment in energetically costly behaviors, such as activity and aggression^7,8^, or risk-taking strategies to secure resources or to meet energetic requirements^9–11^. However, the adoption of these responses is not universal and may vary depending on species-specific traits and ecological contexts. These behavioral changes are closely linked to temperature-induced shifts in metabolic rates^12,13^. Studies have shown that as temperature increases, organisms experience higher standard metabolic rates (minimal metabolic demand to sustain life), reduced maximum metabolic rates (peak metabolic output during exercise) and a narrower aerobic scope (available energy for critical functions like reproduction and growth), as outlined by the Oxygen and Capacity Limited Thermal Tolerance (OCLTT) hypothesis^14,15^.

Likewise, temperature effects on other metabolic parameters such as routine metabolic rates, which reflects the average energy expenditure during normal behaviors, have also been observed^16,17^. During aerobic respiration, the partial reduction of the oxygen (O_2_) molecule leads to the production of reactive oxygen species (ROS), such as hydrogen peroxide and superoxide radicals, which, while essential for maintaining homeostasis and regulating cell signaling^18^, can become harmful when in excess^19^. To counteract the deleterious effects of ROS, marine organisms rely on robust physiological defense mechanisms, including a well-developed antioxidant system^19^. The antioxidant response system involves the action of enzymes such as Superoxide Dismutase (which converts the superoxide anion into hydrogen peroxide and water), Catalase (which converts hydrogen peroxide into O_2_ and water), Glutathione-S-Transferase (which converts xenobiotics into more soluble conjugates), and Glutathione Peroxidase (which converts hydrogen peroxide into water)^20,21^. However, under severe conditions of stress, a cascade of molecular changes may be triggered resulting in enzyme inactivation, lipid peroxidation and DNA damage, by the production and accumulation of ROS to levels that physiological defenses are unable to eliminate, leading to oxidative stress^22^.

While much is known about the physiological aspects of thermal performance, there is a notable gap in holistic studies that integrate physiological, behavioral and biochemical parameters, particularly in the early stages – a critical period characterized by high mortality rates, often due to predation and other environmental pressures^23^. Here we used early life stages (ELS) of *Sparus aurata* (Gilthead seabream) as a model species. This is a commercially important species found in the northeastern Atlantic region (from Cape Verde to the British Isles, Black Sea and Mediterranean)^24^. During spring and summer, larvae move towards coastal nursery areas, such as estuaries^25^, where they grow into juveniles. Consequently, during early developmental stages, the species encounters a broad spectrum of thermal conditions, including both cold extremes and heatwaves documented throughout its distribution range^25^. Early developmental stages are particularly vulnerable to these changes in environmental conditions due to their narrower thermal window and underdeveloped regulatory system^26,27^. Thus, understanding how these early stages respond to temperature variations is vital to predicting population dynamics under climate change scenarios. To address this gap, the present study tested whether long-term exposure to a range of temperatures (19–28 °C) affects thermal performance as measured by body condition, behavior, metabolism and physiology. We also tested whether physiological performance could explain changes in the measured traits to unravel the strategies used by ELS to cope with warming. Additionally, we proposed an integrative approach to evaluate what temperatures represent suitable habitats for ELS by comparing the thermal performance in warmer treatment with that of control condition, which characterizes a cold-end habitat at which the species is adapted.

### Thermal effects on body condition and growth rates

**Fig. 1:**
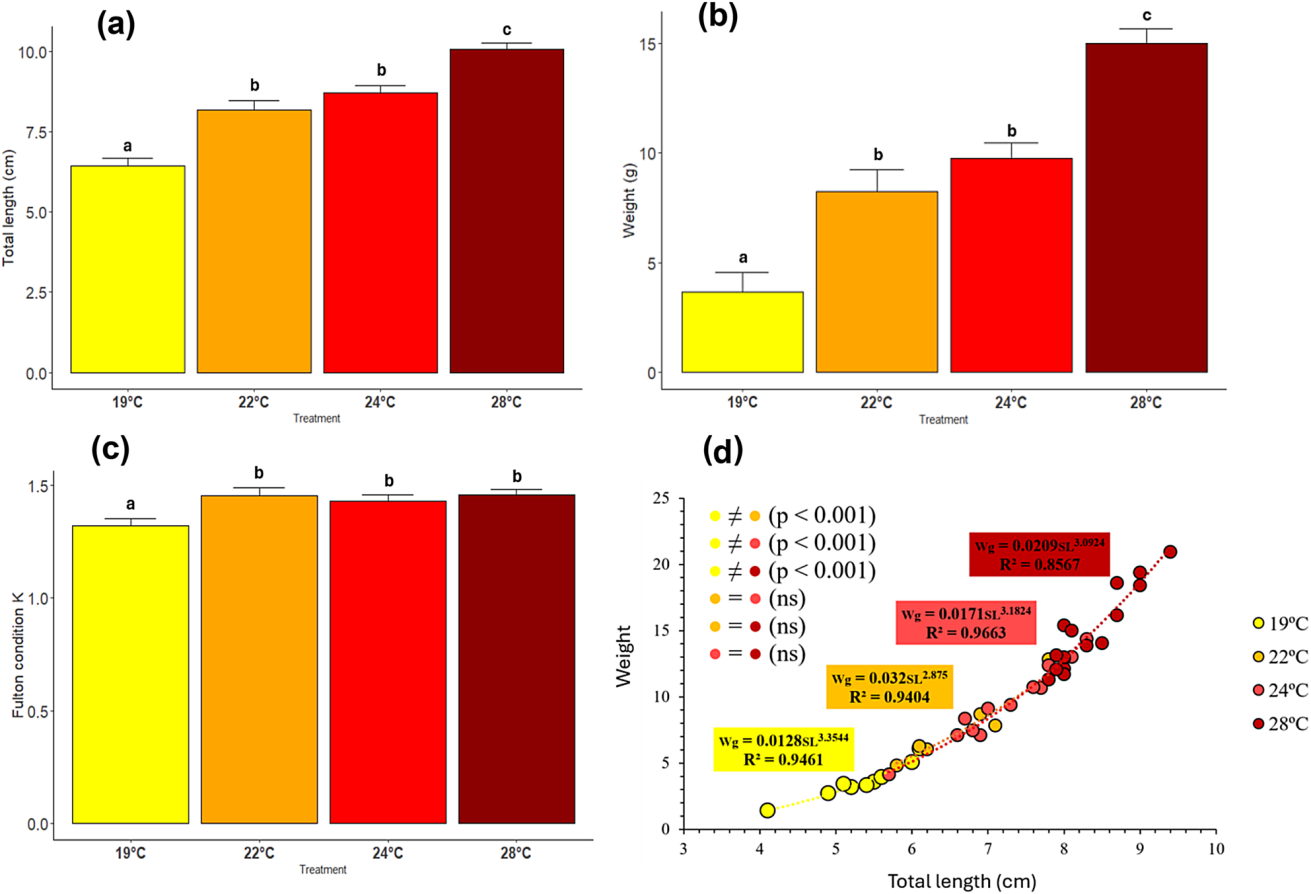
Body condition of *Sparus aurata* exposed to different temperature treatments (19 °C, 22 °C, 24 °C and 28 °C). (**a**) Total length (cm), (**b**) Weight (g), (**c**) Fulton’s condition K and (**d**) allometric growth models. Different letters represent significant differences between treatments. Data expressed as Mean ± s.e.m (total number of individuals tested = 44). ns-non significant.

Total length and weight increased significantly in fish exposed to warmer temperatures (*p* ˂ 0.001, Supplementary Table S1), reaching maximum values of 10.06 ± 0.2 cm and 15 ± 0.68 g (mean ± s.e.m) at 28 °C, respectively (Fig. 1a). Weight reached values > 2.2 to 4 times higher in warmer conditions when compared to 19 °C (3.65 ± 0.87 g) (Fig. 1b). Fulton’s condition (K) did not differ from 22 to 28 °C (ranging from 1.43 ± 0.03 to 1.46 ± 0.03), but increased significantly when compared to 19 °C (1.32 ± 0.04) (*p* = 0.004) (Fig. 1c). ELS gained weight significantly faster (positively allometric) at 19 °C compared to all other treatments (*p* < 0.001) (Fig. 1d). The test treatments did not differ from each other or differ from a isometric slope (Fig. 1d).

### Behavioral responses across different temperatures and exposure period

**Fig. 2:**
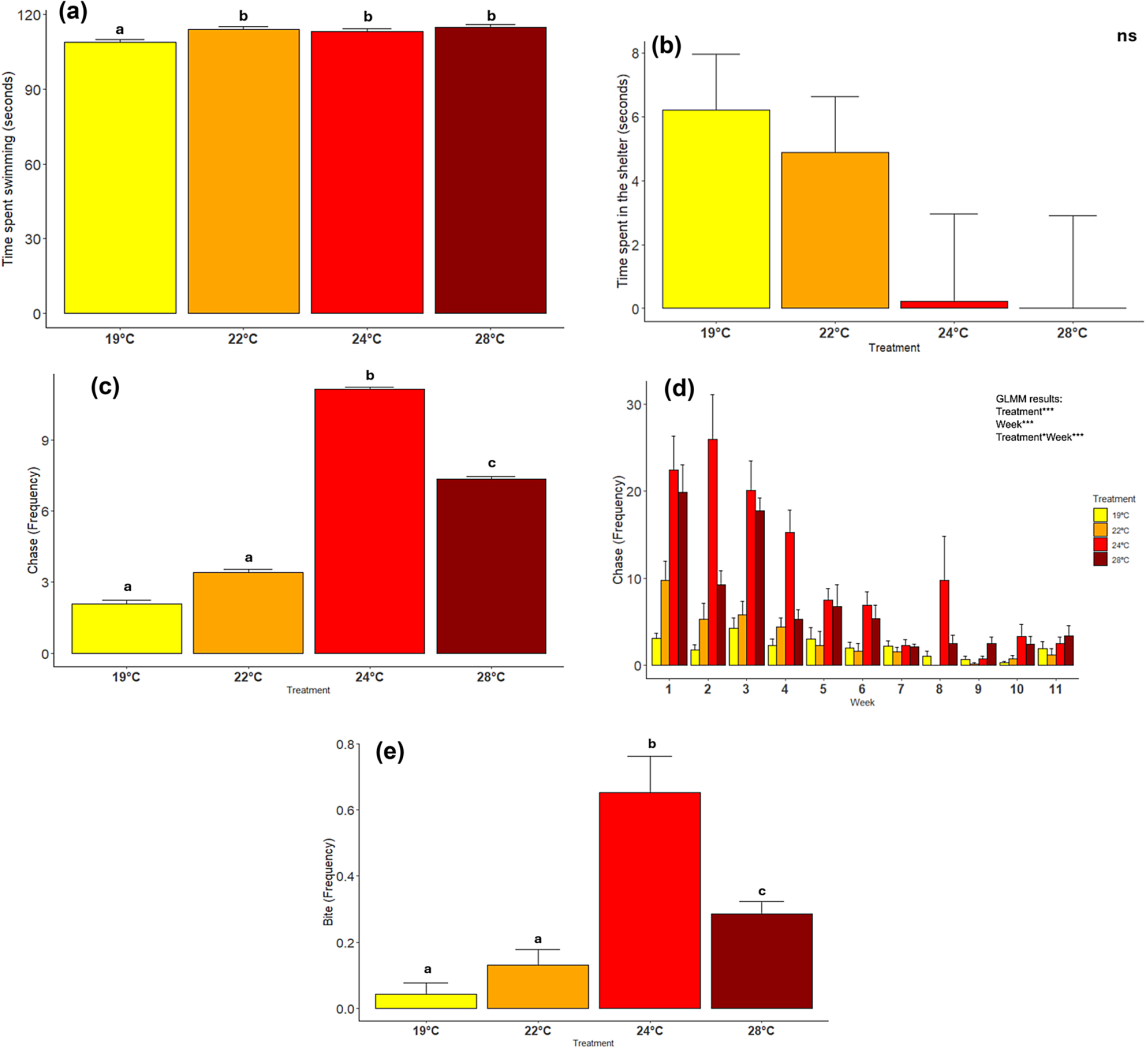
Behavioral responses *Sparus aurata* exposed to different temperature treatments (19 °C, 22 °C, 24 °C and 28 °C). (**a**) time spent swimming (seconds), (**b**) time spent in the shelter (seconds), (**c**) chase (frequency), (**d**) chase over time (frequency) and (**e**) bite (frequency). Different letters represent significant differences between treatments. ns-non significant. Data expressed as Mean ± s.e.m (total number of individuals tested = 367). To check differences in the interaction treatment*week, see Supplementary Tables S3 and S4

The interaction of “treatment” and “exposure time” had no significant effect on swimming activity or the time spent in shelter (Supplementary Table S2). Temperature alone induced changes in swimming activity as larvae spent more time swimming at 22 °C, 24 °C and 28 °C (Fig. 2a). Although there were no differences among elevated temperatures, swimming activity showed a slight but significant increase compared to control (Supplementary Table S2). While temperature had no significant effect on time spent in shelter, a clear trend is observed and, on average, the time spent in shelter decreased with temperature, ranging from 6.22 ± 1.74 s at 19 °C to 0.01 ± 2.88 s at 28 °C (Fig. 2b; Supplementary Table S2). The frequencies of agonistic behaviors (chase and bite) were affected by temperature (chase *p* ˂ 0.001; bite *p* ˂ 0.001; details in Supplementary Table S2). Chase and bites frequencies were significantly higher at 24 and 28 °C, with the highest frequencies being recorded at 24 °C. Chase increased about 4.5 times and bites about 15 times at 24 °C compared to 19 °C (Fig. 2c and e). A significant drop on chase and bites frequencies was also observed at 28 °C compared to 24 °C (chase *p =* 0.013; bite *p =* 0.008). The interaction of “treatment” and “exposure time” had no effect on bite frequency (*p =* 0.974) (Supplementary Table S3). Conversely, the chase frequency was significantly higher at 24 °C until week 4 and at 28 °C until week 3 compared to 19 °C (Supplementary Tables S3 and S4). From week 5 onwards, no significant differences were observed (Fig. 2d) (Supplementary Tables S3 and S4).

### Routine metabolic rates across different temperatures

**Fig. 3:**
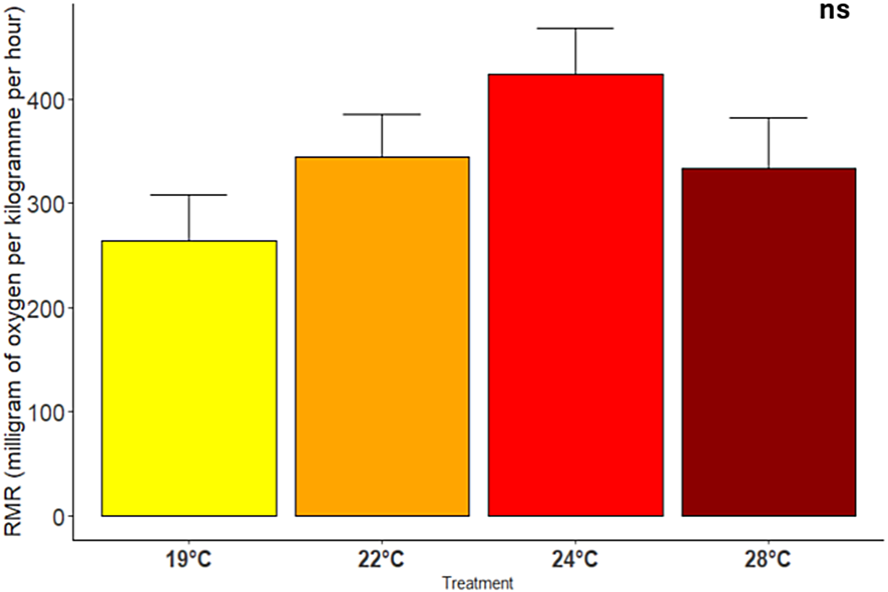
Routine metabolic rate (milligram of oxygen per kilogram per hour) of *Sparus aurata* exposed to different temperature treatments (19 °C, 22 °C, 24 °C and 28 °C). Data expressed as Mean ± s.e.m (total number of individuals tested = 26). ns-non significant.

Routine metabolic rates were not affected by temperature (*p* = 0.252, Supplementary Table S5). However, on average, there was a clear trend of increased oxygen consumption up to 24 °C, with a subsequent reduction at 28 °C (Fig. 3). This pattern was also confirmed by the Q10, which recorded an increase between 22 °C (2.45) and 24 °C (2.59) and a reduction at 28 °C (1.3).

### Thermal effects on molecular responses: aerobic and anaerobic metabolism and oxidative stress

**Fig. 4:**
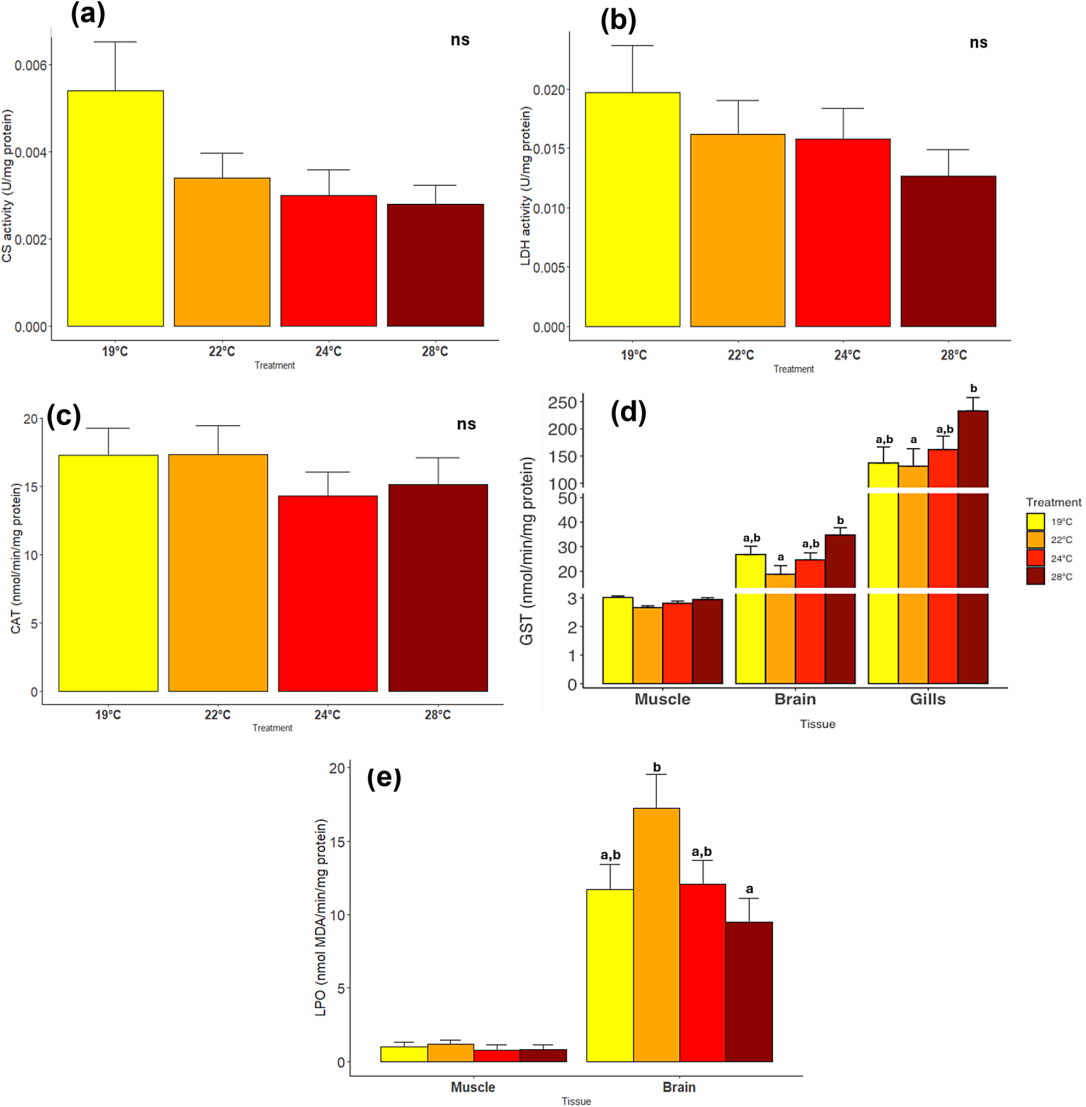
Metabolic enzymes activity and oxidative stress of *Sparus aurata* exposed to different temperature treatments (19 °C, 22 °C, 24 °C and 28 °C): (a) CS (activity per milligram of protein) on muscle, (b) LDH (activity per milligram of protein) on muscle, (c) LPO activity (nmol MDA/mg protein) on muscle and brain, (d) GST (nmol/min/mg protein) on muscle, brain and gills and (e) CAT (nmol/min/mg protein) on gills. Different letters represent significant differences between treatments in the same tissue. ns-non significant. Data expressed as Mean ± s.e.m (total number of individuals tested = 35).

CS and LDH activities were not significantly affected by temperature (CS: *p* = 0.052; LDH: *p* = 0.348), but a clear trend of drop towards higher temperatures was observed (CS varying between 0.0054 ± 0.0011 at 19 °C and 0.0028 ± 0.0004 at 28 °C; and LDH between 0.0197 ± 0.0039 at 19 °C and 0.0127 ± 0.0022 at 28 °C) (Fig. 4a and b). CAT activity in the gills did not differ significantly among treatments (*p =* 0.692, Fig. 4c), ranging from 14.29 ± 1.77 at 19 °C to 17.33 ± 2.12 at 28 °C (values in nmol/min/mg protein). GST activity, on the other hand, increased 1.77-fold in gills (*p* = 0.042), between 22 °C and 28 °C, and 2-fold in the brain (*p* = 0.011, Fig. 4d). In the gills there was also a marginally non-significant increase between 19 °C and 28 °C (*p* = 0.053, Fig. 4d). In contrast, in the muscle, GST levels did not differ significantly among treatments (*p =* 0.956, Fig. 4d), ranging from 2.66 ± 0.06 to 3.02 ± 0.06 nmol/min/mg protein. GST activity was also significantly higher in the gills compared to the muscle and brain (details in Supplementary Table S6). Temperature did not affect LPO in the muscle (*p* = 0.778, details in Supplementary Table S6), but LPO varied significantly in the brain (*p* = 0.02, Fig. 4e). The highest LPO values were observed at 22 °C, while the lowest at 28 °C, even though, these values did not differ from the control treatment (19 °C). The LPO levels were significantly higher in the brain when compared to the muscle (Supplementary Table S6).

### Integrating target traits and physiological responses to unravel coping strategies and the probability of thriving under specific temperature conditions

**Fig. 5:**
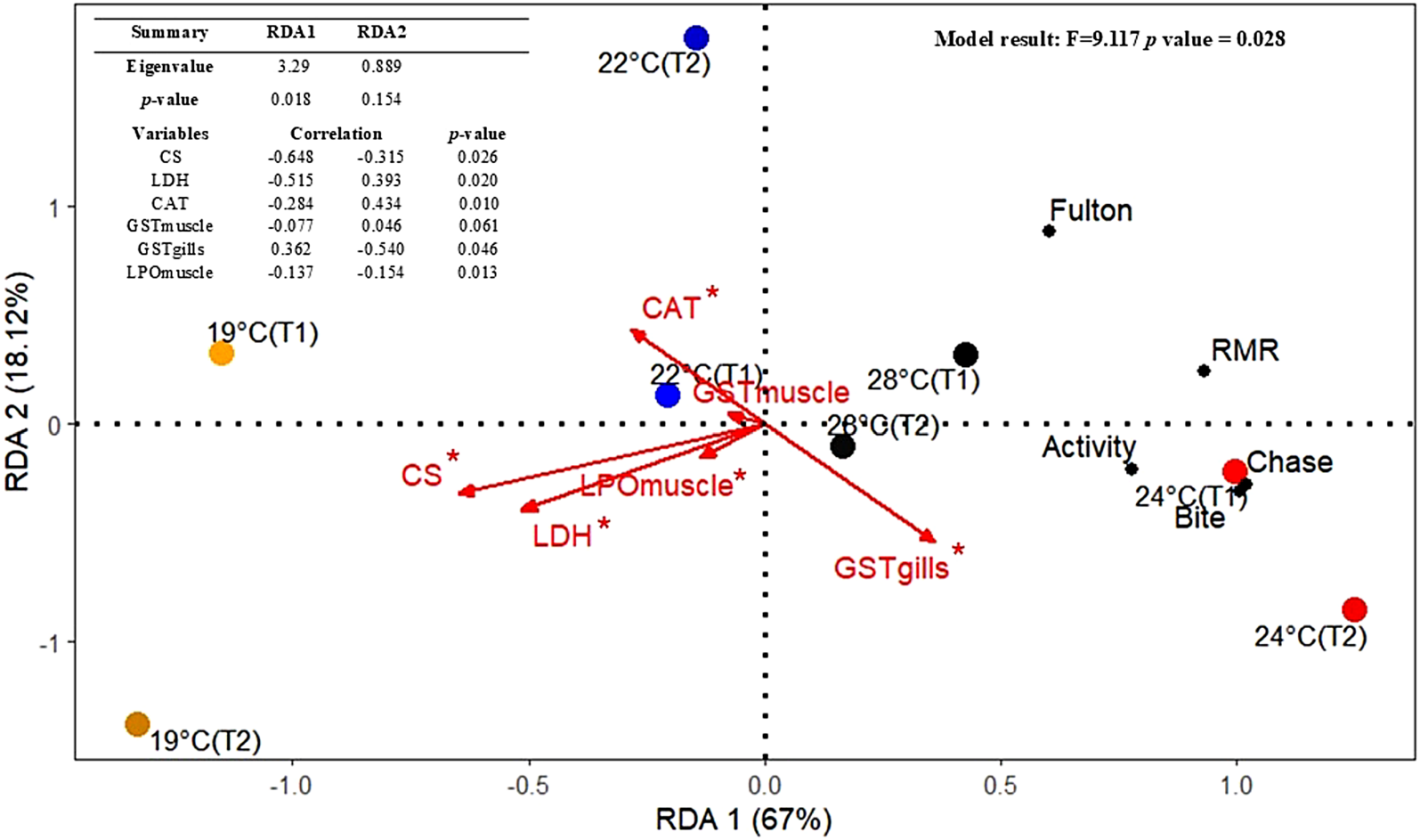
Redundancy analysis (RDA) for the relationships among physiological responses (metabolic and oxidative stress biomarkers) and target traits (Fulton condition K, RMR, activity, chase and bite) after chronic exposure. T1 = tank 1, T2 = tank 2, LDH = Lactate Dehydrogenase, CS = Citrate synthase, CAT = Catalase, LPOmuscle =Lipid Peroxidation in muscle, GSTgills = Glutathione-S-Transferase in gills and GSTmuscle = Glutathione-S-Transferase in muscle. * Represent significant effect.

The RDA analysis showed that metabolic and oxidative stress biomarkers significantly influence the variability in behavior, body condition, and oxygen consumption (*p* = 0.028). The first axis (RDA1) explains 67% of the variation in the relationship between the explanatory and response variables (*p* = 0.018) (Fig. 5). The left portion of the RDA1 is represented by lower temperature treatments and is strongly influenced by CS and LDH activities, while the right portion represents higher temperature treatments highly influenced by gill’s GST activity. RDA1 is positively correlated with GSTgills (0.36) and negatively correlated with CS (-0.65), LDH (-0.52), CAT (-0.28) GSTmuscle (0.08) and LPOmuscle (-0.14). In contrast, the second axis explains only 18.12% of the variability and is not statistically significant (*p* = 0.154). Fulton’s condition, chase, bite, swimming activity and RMR have positive correlation with higher temperature treatments and gill’s GST activity. Conversely, the response variables showed a negative relationship with CS, LDH, CAT, GSTmuscle and LPOmuscle.

**Fig. 6:**
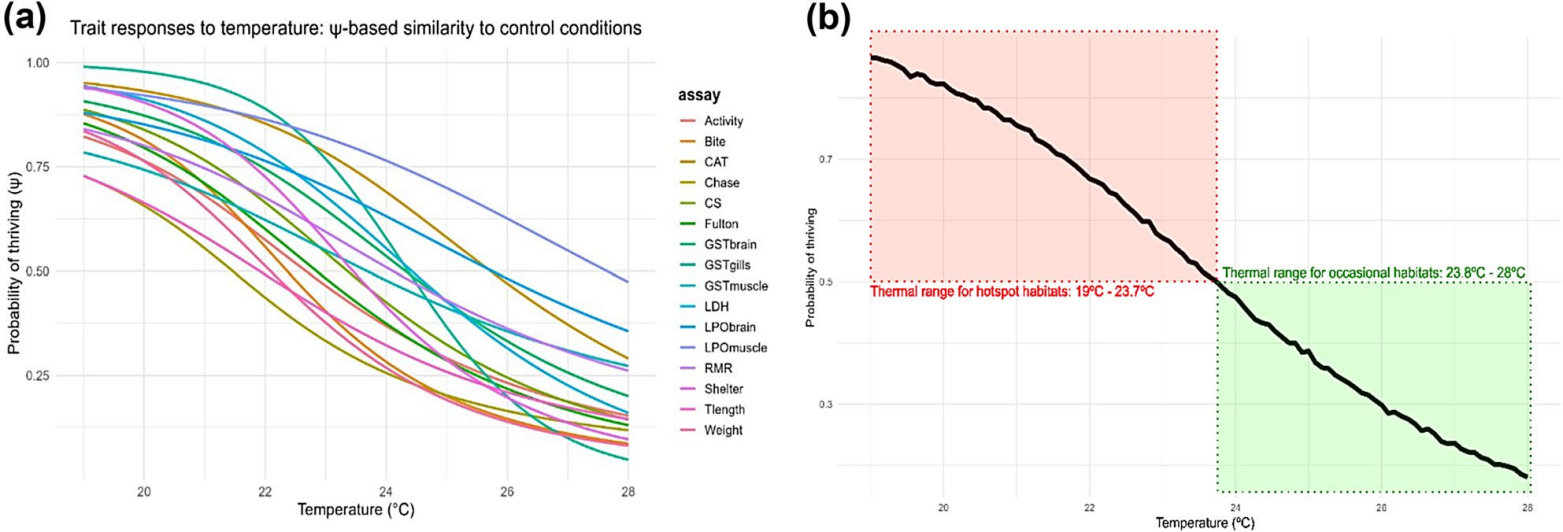
Response curves generated by sub-models. (**a**) Probability of thriving for each trait as a function of temperature calculated from Bayesian probabilities estimations using (**b**) Bayesian GAM integrating JAGS outcomes showing thermal ranges for hotspots and occasional habitats based on the study’s experimental temperatures.

The probability of thriving across a range of temperatures showed that target traits (body condition, behavior and RMR) had > 50% probability of thriving at lower temperatures, as their response curves declined more steeply beyond this range. In contrast, physiological biomarkers (metabolic and oxidative stress indicators) maintained > 50% probability of thriving at higher temperatures, with more gradual declines in their response curves (Fig. 6a). After integrating individual trait in a non-linear model, the result showed that the thermal range securing > 50% chance of thriving (hotspot habitats) is between 19 °C and 23.7 °C; and that securing less than 50% (occasional habitats) is between 23.8 °C and 28 °C (Fig. 6b).

## Discussion

Exposure to temperatures ranging from 22 °C to 28 °C led to increased length and weight and enhanced body condition for *S. aurata* ELS. These finding was somewhat unexpected, as one would assume that fish would enhance consumption of body energy leading to weight and length drops as temperatures exceeded the species’ optimal range. Previous research by Hernández et al. (2003)^28^ identified the optimal growth range for *S. aurata* as being between 24 °C and 26 °C, leading us to anticipate reduced above 26 °C. This decline in growth at higher temperatures has been well-documented in other species, including *Dicentrarchus labrax* (European seabass)^29^, *Salmo trutta* (brown trout)^30^ and *Salmo salar* (Atlantic salmon)^31^. In these cases, growth limitations are likely due to the inability of the circulatory and respiratory systems to supply sufficient oxygen for metabolic needs beyond optimal temperatures^32^. Similarly, reductions in body mass growth rates with rising temperatures have been reported in *Diplodus sargus* (White seabream) and *Trematomus bernacchii* (Emerald rockcod), attributed to decreases in energy reserves, particularly lipid content^33,34^. However, in the present study, *S. aurata* ELS exhibited greater length and weight at higher temperatures, emphasizing that, when food supply is not limited, elevated metabolism is likely to accelerate development to later life stages^35^. This pattern was confirmed by allometric growth models. Fish at elevated temperatures approached the isometric growth, which is a characteristic of later stages, while at 19 °C, smaller fish gained more weight than grew in length. These results suggest that temperature plays a significant role in the growth trajectory of *S. aurata* during the early stages of development. The trend towards isometric growth at higher temperatures suggests ontogenetic acceleration, reflecting faster development under conditions of ample food supply. This pattern is consistent with life-history strategies in which favorable environmental conditions promote rapid growth, potentially increasing survival chances. However, such acceleration may pose an ecological risk^34,35^. If individuals reach recruitment stages before optimal environmental conditions are met, such as suitable habitat availability, access to specific food sources, or reduced predation pressure, a phenological mismatch could compromise their survival and long-term reproductive success^34^. Therefore, while rapid growth may offer short-term advantages, it could also make the species more vulnerable to ecological imbalances driven by climate change^35^.

At the behavioral level, temperature did not significantly influence risk-taking behaviors. However, fish swan for longer periods across the 22 °C, 24 °C and 28 °C treatments. Previous studies showed that activity levels generally follow an asymmetrical curve, peaking at an optimal temperature before declining near the upper thermal limit^7,30^. However, this pattern was not observed in the current study. Fish were able to increase swimming activity at higher temperatures, which is likely due to their ability to maintain stable aerobic and anaerobic metabolism across the range of temperatures, when food is not limited. These signs of thermal response were further supported by changes in agonistic behavior under warmer conditions. Chase and bite frequencies increased with temperature and peaked at 24 °C, suggesting an optimal temperature for agonistic interactions possibly related to territoriality, dominance, or competition for food or space. The subsequent decline at 28 °C may reflect thermal stress or energetic trade-offs^36^. Nevertheless, signs of acclimation were observed after four weeks of exposure to both 24 °C and 28 °C. These patterns have been similarly observed in *Neolamprologus pulcher* (African cichlid), which showed reduced aggression near the species’ thermal limits^8^ and in *Stegastes fuscus* (Brazilian damsel) as it approaches its upper thermal tolerance^37^.

Previous studies have reported positive relationships between agonistic behavior and standard and routine metabolic rates^38,39^. While the mechanisms behind these correlations remain unclear, higher metabolic rates may drive individuals to be more aggressive in securing food and territory^17^. In our study, while oxygen consumption (i.e. RMR) did not differ across different temperatures, a positive correlation between RMR and agonistic behaviors was detected by the RDA model. Therefore, our findings align with previous studies and suggest that, when oxygen is not limiting, aerobic metabolism can be enhanced under warmer conditions to sustain agonistic behavior^40^.

In extreme conditions, the maintenance of RMR close to that observed at lower temperature values could result from shifts in mitochondrial function and protein turnover regulation^33,41^. Similar results were observed in juvenile *S. aurata*, where RMR initially increased with acute exposure to 28 °C, but decreased after two weeks of exposure to values close to 24 °C^42^. Though not statistically significant, the RMR curve in this study mirrors the aerobic scope described by the Oxygen and Capacity Limited Thermal Tolerance (OCLTT) hypothesis proposed by Clark et al. (2013)^43^. Two types of curves have been proposed to characterize the relationship between aerobic performance and temperature: a symmetrical curve^14^ and an asymmetrical curve^44^. Our RMR findings aligns with the asymmetrical curve model proposed by the latter, which assumes that optimal temperature occurs below a peak of aerobic performance, with maximal performance reached near the upper thermal limit, where a shift from aerobic to anaerobic pathways takes place.

Considering these results, expectations would be that LDH activity present in anaerobic pathways, and responsible for the conversion of pyruvate to lactate, would be higher at 28 °C^34,45^. However, LDH did not differ significantly among temperature treatments and even suggested a downward trend. The metabolic biomarkers thus suggest that the RMRs curve resembles the one proposed by Pörtner & Knust (2007)^14^, in which the optimum temperature is located at the peak of aerobic performance, and that the anaerobic pathways are activated at a stage when aerobic performance is closer to zero. Further studies at temperatures above 28 °C may reveal a decrease in aerobic performance until a critical threshold is reached. Previous studies in adults of *S. aurata* reported an increase in LDH activity at 24 °C and limited acclimation above 26 °C, where ATP production declines, leading to increased mortality^46^. The discrepancy between studies likely reflects developmental differences, with juveniles demonstrating greater resilience to thermal stress than adults^25^. The trend of increasing RMRs with temperature was also not accompanied by an increase in aerobic capacity. The RDA shows a negative correlation between RMRs and CS activity, suggesting that while oxygen consumption is greater towards warmer temperatures, uptake by cells is more efficient at colder temperatures. For this species, warming may enhance RMR on average but does not improve aerobic capacity suggesting that thermal stress is more related to oxidative stress. This hypothesis is confirmed by the positive correlation between GST levels in gills and RMRs.

Increased GST activity in brain and gills was observed at 28 °C, supporting the conclusion that enhanced antioxidant defense in warmer conditions led to less oxidative damage since LPO in muscles and in the brain across warmer treatments did not differ from control conditions. However, on average, LPO levels were higher in the muscle compared to the brain. The same was observed for GST where increased levels were found in the muscle, brain and gills, by this order. These results show that muscle is the least susceptible to oxidative stress and gills the most vulnerable, most likely due to greater contact with the external environment^47^. These findings are consistent with Madeira et al. (2016)^47^ who reported similar trends in juvenile *S. aurata* exposed to acute temperature increases, with no significant differences in CAT, GST and LPO in the muscle, brain and gills between 18 °C and 28 °C, with the exception of gill’s GST activity, which increased significantly from 26 °C onwards. This may indicate that ELS are able to maintain their physiology up to 28 °C. These results are also supported by the curves showing the probability of thriving across different temperatures. The curves indicate that *S. aurata* ELS exhibited physiological resilience at warmer temperatures to counterbalance the thermal stress imposed to body condition and behavior.

Overall, *S. aurata* early life stages demonstrate a notable capacity to cope with warming (22–28 °C) through a combination of physiological plasticity, sustained aerobic and anaerobic performance, behavioral adjustments, and activation of antioxidant defenses. Despite expectations of thermal stress and reduced growth above the optimal range (24–26 °C), weight, length and body condition were maintained or even enhanced at 28 °C when food availability enables sufficient energy intake. This suggests that resource availability may be a key modulator of thermal sensitivity. Behaviorally, swimming activity was sustained across temperatures, and agonistic behaviors peaked at 24 °C, declining at 28 °C, possibly reflecting energetic trade-offs or early signs of thermal stress. Interestingly, RMR was positively correlated with agonistic behaviors, indicating that increased metabolism may fuel competitive behaviors if oxygen supply is adequate. At the metabolic level, although not significant, RMR increased on average with temperature and trait-specific probability curves indicate that ELS possess acclimation capacity to elevated temperatures, especially when ecological conditions (e.g., food supply and oxygen availability) are favorable. Despite this, there are signs that ELS may approach physiological limits near or above 28 °C, where aerobic performance plateaus and oxidative stress begin to emerge. Indeed, our integrative approach confirms that it is likely that the species would inhabit habitats with temperatures between 23.8 °C and 28 °C only occasionally, while hotspot habitats are those with temperatures ranging between 19 °C and 23.7 °C. Future increases beyond this threshold may compromise performance and survival.

Future studies should aim to investigate the acute thermal stress imposed by marine heatwaves, as well as the chronic exposure to elevated temperatures simulating climate change, in combination with other environmental and anthropogenic stressors, such as hypoxia, harmful algal bloom toxins and emerging contaminants. This would certainly advance the current knowledge of thermal performance in ELS of commercially and ecologically important fish species.

## Methods

### Ethics

This study was carried out under the approval of Direção Geral de Alimentação e Veterinária (DGAV, Portuguese Authority for Animal Health, permit 6249/25). All methods were performed in accordance with the relevant guidelines and regulations and following the recommendations of the ARRIVE guidelines.

### Fish rearing and experimental setup

Fish trials were conducted between March 13 and June 29, 2023. A total of 440 larvae of *Sparus aurata* (gilthead seabream), at the age of 40 days post-hatch (dph), were transferred from IPMA aquaculture research station (EPPO), in Olhão (Portugal), to ISPA fish facilities. Larvae were transported in containers with controlled temperature and aeration. Spawn was obtained naturally from broodstock reared under aquaculture conditions. At arrival, larvae were placed in a 240-liter quarantine aquarium, equipped with a protein skimmer (TMC Reef skim 200) and mechanical and biological filtration, and enriched with artificial algae. The environmental conditions in the quarantine aquarium - temperature (19 °C), salinity (35 PSU) and photoperiod (14 L:10D) - were maintained to reflect those observed along the Portuguese Atlantic coast at the time. Larvae were left to acclimatize under these conditions for 1 week. During this period, fish were fed *ad libitum* 4 times a day with Winfast 500 feed (SPAROS Lda, Olhão, Portugal, 60% crude protein, 15% crude fat, 12% crude ash). Temperature and salinity were monitored daily.

After acclimation, larvae were transferred to 30-liter aquariums, enriched with PVC pipes that served as shelters, at a density of 22–24 individuals per aquarium. These were equipped with protein skimmers and mechanical and biological filtration systems, with temperature, salinity and photoperiod conditions matching those of the quarantine aquarium. After a 3-day habituation period, the aquariums were randomly assigned to 1 of 4 thermal treatments: (A) 19 °C, corresponding to the temperature experienced by the species along the Portuguese coast during spring/summer [Databases from Instituto Português do Mar e da Atmosfera and Centro de Ciências do Mar e do Ambiente (IPMA)]; (B) 22 °C, representing the projected ocean temperature increase by 2100 according to the IPCC^3^; (C) 24 °C, reflecting the temperature experienced by the species during spring/summer in the nursery zones (IPMA’s databases); and (D) 28 °C, representing temperatures experienced in nursery areas during marine heatwave events^25^, with two replicates per treatment. Temperature in treatments B, C and D was gradually increased at a rate of 1 °C per day using heaters to avoid stress associated with abrupt changes in temperature. The aquariums assigned to the highest temperature (28 °C) were the first to initiate the gradual increase in temperature, followed by the aquariums at 24 °C after 4 days, and the aquariums at 22 °C six days after. This way, it was ensured that all treatments reached their target temperatures simultaneously. Larvae were chronically exposed for 11 weeks to the experimental treatments, during which they were fed ad-libitum twice a day with Winfast 500 (SPAROS Lda, Olhão, Portugal). 11 weeks is considered a long period of exposure, sufficient for acclimation to thermal conditions. Temperature and salinity were measured daily, and other water quality parameters such as nitrites, nitrates and ammonia were analyzed twice a week and kept below critical levels. The excess of food was removed daily from the aquariums to maintain water quality.

Agonistic behaviors, activity levels and risk-taking behaviors were weekly assessed throughout the 11-week exposure period, while routine metabolic rate (RMR) was measured at the end of the experimental period. Subsequently, fish were allowed a one-week recovery period at experimental temperature to mitigate any stress related with handling before being euthanized with an overdose of tricaine methanesulfonate (M222) solution buffered with sodium bicarbonate. The brain, gills and muscle were then extracted for the analysis of biomarkers associated with oxidative stress and metabolic function. Additionally, body weight, total and standard length and the Fulton condition K were determined, with the latter calculated using the following formula:

$$K = 100*(Weight/Lenght^{3} )$$

### Growth patterns

Individuals were randomly collected from each treatment to evaluate the effects of warming on weight growth rates. We used the allometric growth model *Wg* = *β*_0_ S_*L*_^*β*1^ + *ε*, where *Wg* is the weight (dependent variable), S_*L*_ is the standard length (independent variable), *β*_0_ is the intercept and *β*_1_ is the slope or growth coefficient. For isometric growth, the slope *β*_1_ is 3. When the slope *β*_1_ is smaller than 3, the growth is said to be negatively allometric (slower gains in weight); when higher, the growth is said to be positively allometric (faster gains in weight)^48^. To assess whether growth patterns differed among treatments, we compared the estimated slopes by calculating the pairwise differences between slope values and evaluating their significance using the standard errors of the estimates to compute *z*-scores among treatments. Additionally, each slope was tested against the isometric value of 3 using a one-sample *t*-test to determine whether growth within each treatment deviated significantly from the isometric expectation.

### Routine behavior

Behavioral sampling was conducted three times per week over an 11-week period. Focal observations took place between 10:30 and 12:30, approximately 1 h after feeding, to eliminate pre-prandial bias. For that purpose, the observer (always the same) stood in front of each aquarium, and behavioral data collection started once the fish had acclimated to the observer’s presence and resumed their baseline behaviors. Each focal observation lasted 2 min, during which the frequency of conspecific chasing and biting behaviors was recorded (Supplementary Table S7). This was followed by an additional 2-minute period to quantify the time spent swimming and inside the shelters (Supplementary Table S7). This procedure was repeated for 4 individuals per treatment group. To minimize the risk of observing the same individual twice during each observational period, and since no tags were used, we relied on notable differences in fin color, pigmentation and body size.

### Routine metabolic rates

Routine metabolic rates were assessed at the end of the exposure period, using an intermittent-flow respirometry method, as described by Almeida et al. (2024)^49^ and according to the guidelines for reporting methods in aquatic intermittent-flow respirometry^50^. Respirometry trials were conducted between 7:30 and 18:00 h, with two trials per day. Each trial involved operating 8 respirometry chambers simultaneously, divided into two similar set-ups, with each of the 4 chambers connected to a reservoir, allowing us to test two different experimental temperatures at the same time. The water inside the reservoirs had an oxygen concentration of between 6.5 mg/l and 7.5 mg/l. Of these 8 chambers, 6 were designated for fish oxygen consumption, while the remaining 2 served as controls for background respiration. A total of 6–7 fish per treatment were analyzed over 4 days, making a total of 8 runs. Each fish (8.24 ± 4.35 g) was individually placed in an acrylic vacuum-sealed respirometry chamber with a total volume of 315 mL (300 mL empty chamber and 15 mL of tubing). Chambers were submerged in a 55 L water bath maintained at the corresponding temperature treatment. To reduce activity levels associated with inter-individual interaction and external disturbances, the chambers were covered with opaque tape. Following a 1-hour acclimation period, oxygen consumption was measured for 2 h, across three 9.5-minute cycles. Each cycle consisted of a 2-minute measurement period (closed phase), a 30-second waiting period, and a 5-minute flushing period. The duration of each cycle was determined through a preliminary assay to ensure that O_2_ levels inside each chamber did not drop more than 20% (i.e. bellow 80% O_2_ saturation).

**Fig. 7:**
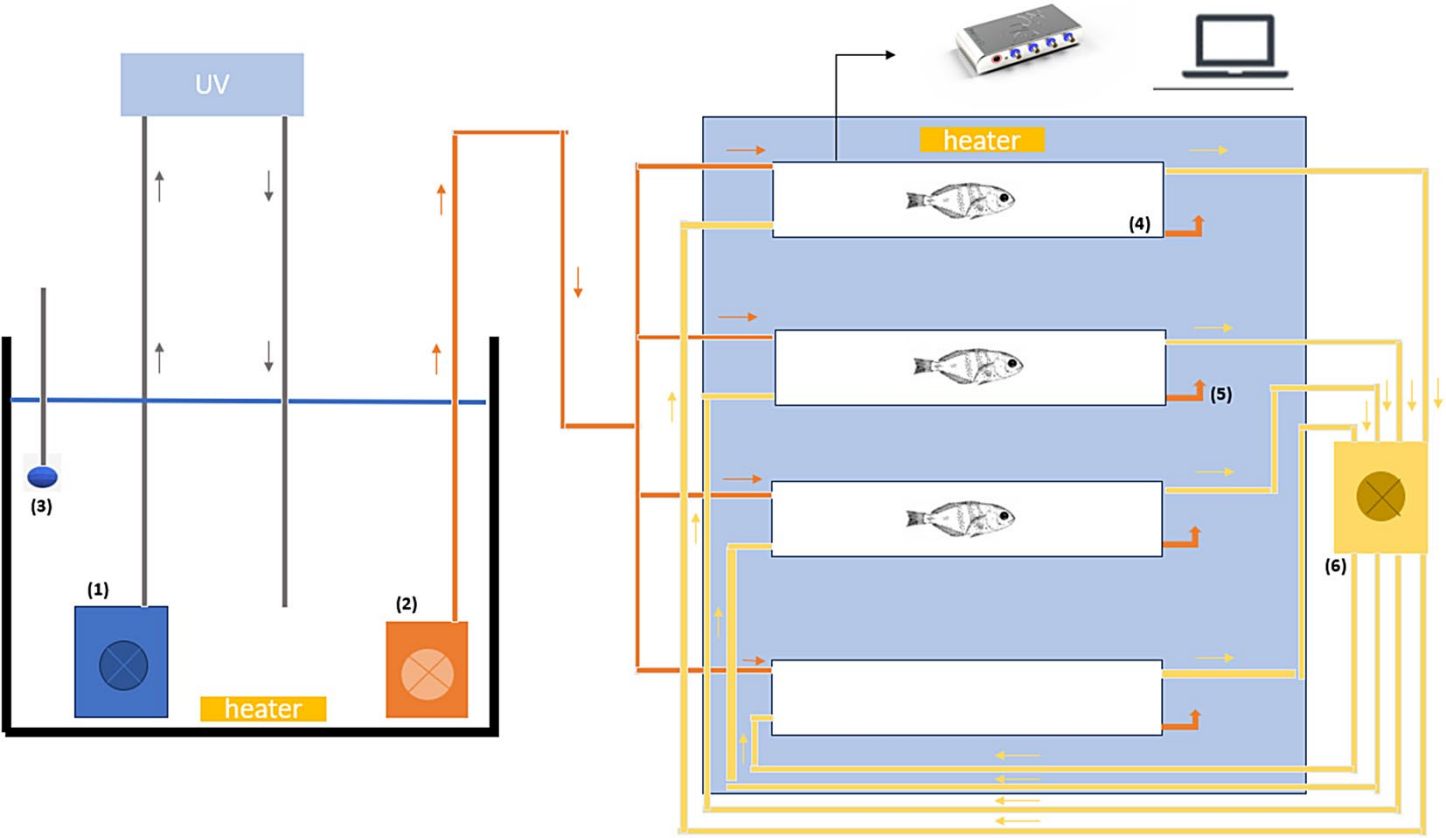
Experimental setup of the intermittent flow system. (1) pump, which will move the water to the UV in order to sterilize it; (2) flush pump, which will move the water to the respirometry chambers during the flush periods; (3) air stone; (4) respirometry chambers; (5) non-return valve, which will prevent the water from returning to the chambers; (6) peristaltic pump, will promote the homogenization of water inside the chambers. Figure adapted from Almeida et al. (2024)^49^.

The flush phase was promoted by a pump controlled via a Profilux controlling system (Profilux4, GHL, Germany), with a programmed timing sequence regulated by the GHL Control Centre Software (version 1.1.4.4). Flush pumps were submerged in a 120 L reservoir, with water matching the temperature and salinity of the original treatment, that allowed the water inside the chamber to be renewed and restored to 100%, at a rate of 236 ml/minute/chamber (Fig. 7). The chambers were connected to a peristaltic pump that allowed water to circulate inside each chamber through a closed external gas-tight piping circuit, with a flow rate of 2 mL/min, to guarantee the mixing of water inside each chamber during the measurement periods. Oxygen inside the chamber was measured using O_2_ sensor spots (OXSP5, Pyroscience, Germany), connected to a Firesting Optical Oxygen Meter (FireSting-O2-4 C, Pyroscience, Germany) via optical fiber cables. The sensors were calibrated, and chambers cleaned with distilled water and 70% ethanol before being refilled with seawater in each run.

The slopes were readjusted to the background and the MO_2_ was calculated for each closed period, considering the weight of the individual and the volume of the system. The volume of the system consisted of the volume of the chamber plus the volume of the connection to the peristaltic pump minus the volume of the individual. The RMR were determined through the mean of MO_2_, and only the values of MO_2_ with R^2^ equal to or greater than 0.9 were considered.

From the RMR data, the thermal sensitivity was analyzed using the following formula:

Q10 = (Rate2/Rate1)^10 / (Temperature2 − Temperature1)^

### Biomarkers analysis

#### Preparation of tissue extracts

Each tissue was homogenized in 0.5-2 mL of phosphate buffered saline (PBS) solution using an Ultra-Turrax device (T25 digital, IKA, Germany). For citrate synthase (CS) and lactate dehydrogenase (LDH), the samples were homogenized in 1 ml of PBS adapted for each enzyme (CS: 20 mM HEPES, 1 mM EDTA and 1% Triton; LDH: 150 mM Imidazole; 1 mM EDTA and 1% Triton). The homogenates were then centrifuged for 15 min at 10,000 *g* at 4 °C. The resulting supernatants were transferred to 1.5 mL microtubes and immediately stored at -40 °C. Total protein quantification was performed using the Bradford method for subsequent normalization of biomarker data^51^. Briefly, 10 µl of each sample was added to a 96-well microplate, along with Bradford reagent, and absorbance read at 595 nm (Thermo Scientific Multiskan GO 1510, Waltham, MA, USA). Protein concentrations were determined by comparing sample absorbance to a calibration curve, created using serial dilutions of bovine serum albumin (BSA) standards ranging from 0 to 1 mg/ml (8 standards in total).

Metabolic enzymes (i.e. CS and LDH) activities were measured in muscle tissues, that plays a central role in locomotion and energy production, as its sensitivity to environmental changes directly affects aerobic and anaerobic metabolic capacities. Antioxidant enzymes activities, such as Catalase (CAT) and Glutathione-S-Transferase (GST), were primarily assessed in gills as they are directly exposed to the external environment, being the first point of contact with environmental disturbances, making them a key tissue for studying the oxidative stress response^52^. Additionally, muscle and brain tissues were also used to assess GST activity and cell damage (i.e. Lipid Peroxidation).

#### Metabolic enzymes

The activity of Citrate Synthase (CS) and Lactate Dehydrogenase (LDH) was determined on muscle, through an adaptation of Rosa et al. (2016)^53^. Briefly, CS was quantified by adding 10 µl of sample, 200 µl of reaction buffer (0.25 mM of DTNB and 75 mM of Tris), 10 µl of acetyl-CoA and 10 µl of oxalocetic to each well of a microplate. The plate was left to incubate for 2 min at room temperature and then the absorbance was read at 412 nm (Thermo Scientific Multiskan GO 1510, Waltham, MA, USA), every minute for 5 min. LDH activity was analyzed by adding 5 µl of sample, 200 µl of reaction buffer (0.15 mM of NADH; 1 mM of KCN and 1mM of Imididazol) and 10 µl of pyruvic acid to each well of the microplate. After a period of incubation of 2 min at room temperature, the absorbance was read at 340 nm (Thermo Scientific Multiskan GO 1510, Waltham, MA, USA), every minute for 5 min.

#### Antioxidant enzymes

Catalase (CAT) activity was measured in the gills following the method described by Johansson & Borg (1988)^54^. Briefly, 20 µL of sample, 100 µL of potassium phosphate (100 mM), 30 µL of methanol and 20 µL of hydrogen peroxide were added to a 96-well microplate. The microplate was allowed to incubate for 20 min, with continued shaking. Subsequently, 30 µL of potassium hydroxide (10 M) and 30 µL of purpald solution (0.5 M) were added to each well, and the microplate was incubated for another 10 min with shaking. Finally, 10 µL of potassium periodate solution (65.2 mM in 0.5 M potassium hydroxide) was added into each well and the absorbance was measured at 540 nm (Thermo Scientific Multiskan GO 1510, Waltham, MA, USA). Catalase activity was quantified using a formaldehyde calibration curve (0–75 mM), where one unit of catalase was defined as the amount of enzyme required to produce 1 nmol of formaldehyde per minute at 25 °C.

Glutathione-S-Transferase (GST) levels were quantified in muscle, brain and gills according to Habig et al. (1974)^55^. In brief, 20 µL of sample and 180 µL of reaction mix [200 mM reduced L-glutathione, 100 mM 1-chloro-2,4- dinitrobenzene (CDNB) and Dulbeco buffer (Sigma-Aldrich)] were added to a 96-well microplate. The absorbance was read at 340 nm every minute for 6 min (Thermo Scientific Multiskan GO 1510, Waltham, MA, USA). GST activity was calculated based on the absorbance increase per minute, using the CDNB extinction coefficient (5.3 mM^− 1^ cm^− 1^).

#### Oxidative damage

Lipid Peroxidation (LPO) was analyzed in muscle and brain using the TBARS method^56^, by quantifying malondialdehyde (MDA), an end product of lipid peroxidation. Briefly, 50 µL of sample and 250 µL of mix solution [sodium dodecyl sulphate (8.1%), trichloroacetic acid (20%, pH 3.5), thiobarbituric acid (1%), and Milli Q water] were added to each microtube. Subsequently, each microtube was mixed and placed in a water bath (100 °C) for 10 min. After this period, the microtubes were placed on ice to stop the reaction and 62.5 µL Milli-Q water was added. Finally, the microtubes were centrifuged at 5000 g for 5 min, 100 µL of supernatant was removed and placed in a microplate and the absorbance read at 532 nm (Thermo Scientific Multiskan GO 1510, Waltham, MA, USA). The MDA concentration was determined based on a calibration curve (0-0.3 µM MDA bis) and using extinction coefficient (155 mM^− 1^ cm^− 1^).

### Bayesian probability estimation with JAGS: mechanistic sub-models

Separate sub-models were developed for each response type—body condition, physiological, and behavioral. These responses were converted into probabilities reflecting the likelihood that ELS would thrive under specific environmental scenarios (i.e., experimental treatments). The underlying assumption is that ELS are most likely to thrive (*ψ* = 1) when the average value of a given trait under a test condition aligns with that observed in the control treatment. Nonetheless, due to natural variability and measurement error, trait values in test conditions rarely match the control exactly, leading to predicted probabilities of presence (*ψ*) ranging between 0 and 1 depending on temperature increases. The threshold for each trait was defined by the mean value observed in the control. To convert trait responses into probability estimates, Bayesian beta regression was applied using JAGS, version 3.4.0^57^. For each trait, a z-score was calculated relative to the control treatment and transformed via the logistic function:

$$\:Z=\:\frac{X-\:{\mu\:}_{Control}}{\sqrt{{SE}_{x}^{2}}+{SE}_{Control}^{2}}$$

where *X* represents the mean value of a given treatment, *µ*_Control_ is the mean of the control group, and *SE* represents the standard error. The probability metric was computed as:

$$\:\psi\:=\text{e}\text{x}\text{p}(-\left|\text{Z}\right|)$$

ensuring that values remained within the (0,1) interval using a small threshold ϵ (10^− 6^). The response variable *ψ*_*i*_ (for the *i*-th measurement) was assumed to follow a Beta distribution:

$$\:{\psi\:}_{i}\:\sim\:\text{B}\text{e}\text{t}\text{a}({p}_{i},\:{q}_{i})$$

Where *p*_*i*_ and *q*_*i*_ are parameterized as:

$$p_{i} = ~\mu _{i} ~\cdot~~\phi ,~~q_{i} = \left( {1 - ~\mu _{i} } \right)~\cdot~~\phi$$

The linear predictor incorporated the effects of temperature to match the same experimental setup:

$$\:logit\left({\mu\:}_{i}\right)={a}_{0}+{a}_{1}{TEMP}_{i}$$

The model estimates the probability of thriving (*ψ*) by integrating the Beta probability density function from 0 to 1, using a Bayesian beta-regression approach. The Beta distribution’s mean and standard deviation correspond to the observed average and variability of each treatment. For traits where higher values relative to the control indicate reduced fitness (e.g., LPO and LDH activity), the inverse of the Z-score was used to correct directionality. Weakly informative normal priors were assigned to each coefficient of the parameter *k* (i.e. *intercept*,* TEMP*).

$$\:{a}_{k}\:\sim\:Normal\left(0,\:0.001\right),\:\:k=\text{0,1},\text{2,3}$$

The precision parameter *ϕ* followed an uninformative prior^58^:

$$\:\varphi\:={U}^{2},\:\:\text{U}\:\sim\:\text{U}\text{n}\text{i}\text{f}\text{o}\text{r}\text{m}(0,\:50)$$

Three Markov Chain Monte Carlo (MCMC) chains, with 1000 tuning iterations, 4000 burn-in iterations, and 2000 post-burn-in samples were used to run the model Gelman-Rubin diagnostics was used to assess convergence^58^.

Each treatment’s specific temperature allowed the model to compute the relationship between the predicted probability (*ψ*_*B*_​) as a function of a broader range of temperatures (19–28 °C) for each trait^59^.

### Bayesian generalized additive model (GAM) for integrated estimation

The trait-specific predicted probabilities (ψ) were combined into a unified model. Since each sub-model provides a distinct probability of thriving at a given temperature, this integration captures the idea that multiple traits may interact, potentially in non-linear ways, to influence organismal performance. To explore these relationships, we applied a Bayesian Generalized Additive Model (GAM) using the *brms* package with default prior settings. The model structure was:

$$\:\psi\:\:\sim\:\text{s}\left(TEMP\right)$$

where *s(TEMP)* denotes a smooth term modeled with penalized splines. A Beta distribution with a logit link function was used to accommodate the bounded nature of the response variable. The resulting model produces a response curve depicting how predicted probabilities vary across the temperature gradient.

### Data analysis

Prior to model fitting, histograms and the probability distributions of the response variables were examined to select the appropriate distribution family for each measured trait, using the fitdistrplus R package. The effect of temperature on weight, total length, Fulton’s condition (K-index) and routine metabolic rates were analyzed through a linear mixed effect models (“lmerTest” R package), following a normal distribution. The effects of the interaction between treatments vs. time of exposure (or weeks) were assessed on behavioral responses using generalized linear mixed models (GLMM, “glmmTMB” R package). Chase and bite were modelled through the Tweedie distribution (link function: “Log”). The time spent swimming and in the shelter were analyzed using a Poisson distribution (link function: “Log”). The effect of temperature over time on these variables was analyzed using “treatments” and “weeks” as fixed interacting factors, and the “aquarium” as a random factor. GLMMs were also used to test the effect of “treatment” on molecular biomarkers. The levels of LPO in the muscle, GST in the muscle and in the gills, as well as GST levels between tissues were modelled through the Gamma distribution (link function: “Log”). The LPO levels between tissues, LDH and CS activity were analyzed using lognormal distribution (i.e. a normal distribution with a log link function). The levels of LPO and GST in the brain were analyzed using linear mixed effect models through a normal distribution. For these models, “treatment” was considered a fixed factor and “aquarium” as random factor. For comparisons of GST and LPO levels between tissues, “tissue” was treated as the fixed factor. Whenever significant differences were detected, the Tukey post hoc test was used to find the sources of variance among the tested factors.

Redundancy Analysis (RDA) was used to assess whether the effects of temperature on measured traits, namely body condition, behavior (chasing, biting, and activity) and RMRs, could be explained by metabolic and oxidative stress biomarkers. A matrix containing the measured traits for each group (i.e., different treatments and aquarium) served as the response variable, while a matrix of variables related to changes in metabolic and oxidative stress was used as the explanatory set. Before running the model, the response variables were transformed through the “standardize” method using the decostand function, while the explanatory variables were log (x + 1) transformed. The overall statistical significance of each RDA model, the constrained axes, and individual variables were evaluated using a permutation-based ANOVA test. Explanatory variables selected through stepwise procedures are represented as vectors radiating from the origin in the ordination plot. All statistical analysis was developed in R statistics, Rstudio (Version 4.2.2). Values are reported as means ± standard error (s.e.m), and P values below 0.05 were considered significant.

## Supplementary Information

Below is the link to the electronic supplementary material.

Supplementary Material 1

## Data Availability

Data available upon request from the corresponding author, João Carlos Almeida.
