# Supplementary material for: Thermal performance of early stages of Sparus aurata integrating body condition, behavior and physiological responses
Source: Sci Rep. 2025 Nov 6;15:38852. doi: 10.1038/s41598-025-22781-x (PMC12592365; doi:10.1038/s41598-025-22781-x)
Supplement: Supplementary file 1 — Supplementary Material 1 [file 41598_2025_22781_MOESM1_ESM.docx]

**Thermal performance of early stages of *Sparus aurata* integrating body condition, behavior and physiological responses**

*João Carlos Almeida^1^, Ana Beatriz Costa^2^, Buzenur Ozkan^3^, Sara Martins-Cardoso ^1^, Ana Luísa Maulvault^2,4,5^, Pedro Pousão-Ferreira^3^, Laura Ribeiro^6^, André Ricardo Araújo Lima^7,8^, Ana Margarida Faria^2,9^, Ana Rita Lopes^2,10^

*****Corresponding author: joaocarlos111997@gmail.com

**Authors affiliation**

^1^ MARE - Marine and Environmental Sciences Centre/ARNET-Aquatic Research Network, ISPA-Instituto Universitário, 1149-041 Lisbon, Portugal

^2^ MARE - Marine and Environmental Sciences Centre/ARNET-Aquatic Research Network, Faculty of Sciences, University of Lisbon, Campo Grande, 1749-016 Lisbon, Portugal

^3^ IPMA, I.P., Portuguese Institute for the Sea and Atmosphere, I.P., Division of Aquaculture, Upgrading and Bioprospection, Av. Doutor Alfredo Magalhães Ramalho 6, 1495-165 Lisbon, Portugal

^4^ UCIBIO — Applied Molecular Biosciences Unit, NOVA School of Science and Technology, NOVA University of Lisbon, Campus de Caparica, 2829-516 Caparica, Portugal

^5^ Associate Laboratory i4HB Institute for Health and Bioeconomy, NOVA School of Science and Technology, NOVA University of Lisbon, 2829-516 Caparica, Portugal

^6^ Faculdade de Ciências e Tecnologia, Universidade do Algarve, Campus de Gambelas, 8005-139 Faro, Portugal

^7^CIBIO, Centro de Investigação em Biodiversidade e Recursos Genéticos, InBIO Laboratório Associado, Universidade do Porto, Vairão, Portugal

^8^BIOPOLIS Program in Genomics, Biodiversity and Land Planning, CIBIO, Vairão, Portugal

^9^Atlantic International Research Centre, Lisbon, Portugal

^10^ Department of Animal Biology, Faculty of Sciences, University of Lisbon, Campo Grande, 1749-016 Lisbon, Portugal.

**Supplementary material**

**Table S1-** LMM analysis. Effects of Treatment on weight, total length and Fulton's condition K. Comparisons between treatments. Est-Estimates, Std error-Standard error.

| Weight | | | | |
| --- | --- | --- | --- | --- |
|  | chisq | p-value |  |  |
| Treatment | 111.08 | ˂0.001 |  |  |
|  | Est | SE | z-value | p-value |
| 19°C-22°C | 4.6 | 1.318 | 3.489 | 0.003 |
| 19°C-24°C | 6.087 | 1.134 | 5.367 | ˂0.001 |
| 19°C-28°C | 11.348 | 1.103 | 10.29 | ˂0.001 |
| 22°C-24°C | 1.488 | 1.226 | 1.213 | 0.616 |
| 22°C-28°C | 6.747 | 1.197 | 5.637 | ˂0.001 |
| 24°C-28°C | 5.261 | 0.991 | 5.308 | ˂0.001 |
| Total length | | | | |
|  | chisq | p-value |  |  |
| Treatment | 130.47 | ˂0.001 |  |  |
|  | Est | SE | z-value | p-value |
| 19°C-22°C | 1.764 | 0.385 | 4.585 | ˂0.001 |
| 19°C-24°C | 2.286 | 0.331 | 6.906 | ˂0.001 |
| 19°C-28°C | 3.638 | 0.322 | 11.305 | ˂0.001 |
| 22°C-24°C | 0.522 | 0.358 | 1.459 | 0.46 |
| 22°C-28°C | 1.874 | 0.349 | 5.365 | ˂0.001 |
| 24°C-28°C | 1.352 | 0.289 | 4.676 | ˂0.001 |
| Fulton's condition K | | | | |
|  | chisq | p-value |  |  |
| Treatment | 13.376 | 0.004 |  |  |
|  | Est | SE | z-value | p-value |
| 19°C-22°C | 0.134 | 0.048 | 2.803 | 0.026 |
| 19°C-24°C | 0.111 | 0.041 | 2.695 | 0.035 |
| 19°C-28°C | 0.137 | 0.4 | 3.431 | 0.003 |
| 22°C-24°C | -0.023 | 0.045 | -0.52 | 0.954 |
| 22°C-28°C | 0.003 | 0.043 | 0.074 | 0.999 |
| 24°C-28°C | 0.026 | 0.036 | 0.733 | 0.883 |

**Table S2-** GLMM analysis. Effects of Treatment on activity levels, time spent in the shelter, chase and bite. Comparisons between treatments. Est-Estimates, Std error-Standard error.

| Activity levels | | | | |
| --- | --- | --- | --- | --- |
|  | chisq | p-value |  |  |
| Treatment | 13.534 | 0.004 |  |  |
| Treatment*Week | 66.314 | 0.076 |  |  |
|  | Est | Std. Error | z-value | p-value |
| 19°C-22°C | -4.556 | 1.22 | -3.735 | 0.001 |
| 19°C-24°C | -6.115 | 1.24 | -4.951 | ˂0.001 |
| 19°C-28°C | -5.397 | 1.23 | -4.371 | ˂0.001 |
| 22°C-24°C | -1.559 | 1.21 | -1.292 | 0.569 |
| 22°C-28°C | -0.841 | 1.20 | -0.699 | 0.898 |
| 24°C-28°C | 0.717 | 1.21 | 0.592 | 0.935 |
| Time spent in the shelter | | | | |
|  | chisq | p-value |  |  |
| Treatment | 5.044 | 0.169 |  |  |
| Treatment*Week | 44.776 | 0.103 |  |  |
|  | Est | Std. Error | z-value | p-value |
| 19°C-22°C | 0.242 | 0.367 | 0.660 | 0.912 |
| 19°C-24°C | 3.321 | 3.158 | 1.052 | 0.719 |
| 19°C-28°C | 6.046 | 3.275 | 1.846 | 0.252 |
| 22°C-24°C | 3.079 | 3.163 | 0.974 | 0.765 |
| 22°C-28°C | 5.804 | 3.28 | 1.77 | 0.288 |
| 24°C-28°C | 2.725 | 3.784 | 0.72 | 0.889 |
| Chase | | | | |
|  | chisq | p-value |  |  |
| Treatment | 136.346 | ˂0.001 |  |  |
| Treatment*Week | 71.847 | ˂0.001 |  |  |
|  | Est | Std. Error | z-value | p-value |
| 19°C-22°C | 0.611 | 0.118 | -2.542 | 0.054 |
| 19°C-24°C | 0.188 | 0.032 | -9.737 | ˂0.001 |
| 19°C-28°C | 0.284 | 0.051 | -7.055 | ˂0.001 |
| 22°C-24°C | 0.307 | 0.048 | -7.574 | ˂0.001 |
| 22°C-28°C | 0.464 | 0.076 | -4.700 | ˂0.001 |
| 24°C-28°C | 1.514 | 0.207 | 3.027 | 0.013 |
| Bite | | | | |
|  | chisq | p-value |  |  |
| Treatment | 21.730 | ˂0.001 |  |  |
| Treatment*Week | 16.883 | 0.974 |  |  |
|  | Est | Std. Error | z-value | p-value |
| 19°C-22°C | 0.3333 | 0.19 | -1.903 | 0.227 |
| 19°C-24°C | 0.067 | 0.035 | -5.233 | ˂0.001 |
| 19°C-28°C | 0.137 | 0.073 | -3.729 | 0.001 |
| 22°C-24°C | 0.201 | 0.064 | -5.071 | ˂0.001 |
| 22°C-28°C | 0.410 | 0.1409 | -2.594 | 0.047 |
| 24°C-28°C | 2.041 | 0.462 | 3.154 | 0.008 |

**Table S3-** GLMM analysis. Effects of Treatment on chase over the 11 weeks of exposure. Comparisons between treatments, for each week.

|  | 19°C | 22°C | 24°C | 28°C |
| --- | --- | --- | --- | --- |
| 19°C week1 |  | z-value -3,042  *p*-value 0,5295 | z-value -6,145  *p*-value ˂0.0001 | z-value -5,728  *p*-value ˂0.0001 |
| 22°C week1 |  |  | z-value -3,674  *p*-value 0,1173 | z-value -3,161  *p*-value 0,4275 |
| 24°C week1 |  |  |  | z-value 0,514  *p*-value 1 |
| 28°C week1 |  |  |  |  |
| 19°C week2 |  | z-value -1,819  *p*-value 0,9997 | z-value -6,145  *p*-value ˂0.0001 | z-value -3,546  *p*-value 0,1709 |
| 22°C week2 |  |  | z-value -5,247  *p*-value 0.0001 | z-value -1,953  *p*-value 0,9986 |
| 24°C week2 |  |  |  | z-value 3,833  *p*-value 0,0704 |
| 28°C week2 |  |  |  |  |
| 19°C week3 |  | z-value -0, 726  *p*-value 1 | z-value -4,653  *p*-value 0.0026 | z-value -4,328  *p*-value 0.0109 |
| 22°C week3 |  |  | z-value -4,183  *p*-value 0.0196 | z-value -3,825  *p*-value 0.0724 |
| 24°C week3 |  |  |  | z-value 0,436  *p*-value 1 |
| 28°C week3 |  |  |  |  |
| 19°C week4 |  | z-value -1, 664  *p*-value 1 | z-value -5,528  *p*-value ˂0.0001 | z-value -2,212  *p*-value 0.9855 |
| 22°C week4 |  |  | z-value -4,384  *p*-value 0.0086 | z-value -0, 578  *p*-value 1 |
| 24°C week4 |  |  |  | z-value 3, 937  *p*-value 0,0493 |
| 28°C week4 |  |  |  |  |
| 19°C week5 |  | z-value 0,761  *p*-value 1 | z-value -1, 694  *p*-value 0,9999 | z-value -1, 471  *p*-value 1 |
| 22°C week5 |  |  | z-value -2,203  *p*-value 0,9865 | z-value -2,023  *p*-value 0,9971 |
| 24°C week5 |  |  |  | z-value 0,253  *p*-value 1 |
| 28°C week5 |  |  |  |  |
| 19°C week6 |  | z-value 0,892  *p*-value 1 | z-value -2,590  *p*-value 0,8740 | z-value 1,850  *p*-value 1 |
| 22°C week6 |  |  | z-value -3,095  *p*-value 0,4834 | z-value -2,492  *p*-value 0,9197 |
| 24°C week6 |  |  |  | z-value 0,810  *p*-value 1 |
| 28°C week6 |  |  |  |  |
| 19°C week7 |  | z-value 0,112  *p*-value 1 | z-value -0,292  *p*-value 1 | z-value 0,899  *p*-value 1 |
| 22°C week7 |  |  | z-value -0,909  *p*-value 1 | z-value -1,309  *p*-value 1 |
| 24°C week7 |  |  |  | z-value -0,402  *p*-value 1 |
| 28°C week7 |  |  |  |  |
| 19°C week8 |  | z-value 0,001  *p*-value 1 | z-value -2,248  *p*-value 0,9812 | z-value -1,115  *p*-value 1 |
| 22°C week8 |  |  | z-value -0,001  *p*-value 1 | z-value -0,001  *p*-value 1 |
| 24°C week8 |  |  |  | z-value 1,420  *p*-value 1 |
| 28°C week8 |  |  |  |  |
| 19°C week9 |  | z-value 1,095  *p*-value 1 | z-value -0,580  *p*-value 1 | z-value -1,115  *p*-value 0,9990 |
| 22°C week9 |  |  | z-value -1,532  *p*-value 1 | z-value -2,396  *p*-value 0,9517 |
| 24°C week9 |  |  |  | z-value -1,518  *p*-value 1 |
| 28°C week9 |  |  |  |  |
| 19°C week10 |  | z-value -0,977  *p*-value 1 | z-value -2,294  *p*-value 0,9742 | z-value -2,107  *p*-value 0,9938 |
| 22°C week10 |  |  | z-value -1,610  *p*-value 1 | z-value -1,369  *p*-value 1 |
| 24°C week10 |  |  |  | z-value 0,278  *p*-value 1 |
| 28°C week10 |  |  |  |  |
| 19°C week11 |  | z-value 0,831  *p*-value 1 | z-value -1,067  *p*-value 1 | z-value -1,209  *p*-value 1 |
| 22°C week11 |  |  | z-value -1,825  *p*-value 0,9997 | z-value -1,940  *p*-value 0,9988 |
| 24°C week11 |  |  |  | z-value -0,182  *p*-value 1 |
| 28°C week11 |  |  |  |  |

**Table S4-** GLMM analysis. Effects of Treatment on chase over the 11 weeks of exposure. Comparisons between weeks, for each treatment.

| 19°C | |  |  |  |  |  |  |  |  |  |  |
| --- | --- | --- | --- | --- | --- | --- | --- | --- | --- | --- | --- |
|  | week1 | week2 | week3 | week4 | week5 | week6 | week7 | week8 | week9 | week10 | week11 |
| week1 |  | z-value 1.178  *p-*value  1.0000 | z-value -0.530  *p-*value 1.0000 | z-value 0.999  *p-*value 1.0000 | z-value 0.147  *p-*value 1.0000 | z-value 0.818  *p-*value 1.0000 | z-value 0.968  *p-*value 1.0000 | z-value 1.431  *p-*value 1.0000 | z-value 2.386 *p-*value 0.9545 | z-value 2.717  *p-*value 0.7964 | z-value 1.436  *p-*value 1.0000 |
| week2 |  |  | z-value -1.543 *p-*value 1.0000 | z-value -0.307 *p-*value 1.0000 | z-value -0.770 *p-*value 1.0000 | z-value -0.363  *p-*value 1.0000 | z-value  -0.336  *p-*value 1.0000 | z-value 0.635  *p-*value 1.0000 | z-value  1.399  *p-*value 1.0000 | z-value  -1.893  *p-*value 0.9993 | z-value  -1.761  *p-*value 0.9999 |
| week3 |  |  |  | z-value 1.422  *p-*value 1.0000 | z-value 0.517  *p-*value 1.0000 | z-value 1.222  *p-*value 1.0000 | z-value 1.394  *p-*value 1.0000 | z-value 1.679  *p-*value 1.0000 | z-value 2.634  *p-*value 0.8492 | z-value  -2.925  *p-*value 0.6315 | z-value  -1.761  *p-*value 0.9999 |
| week4 |  |  |  |  | z-value -0.569  *p-*value 1.0000 | z-value -0.084  *p-*value 1.0000 | z-value  -0.032  *p-*value 1.0000 | z-value 0.870  *p-*value 1.0000 | z-value 1.718  *p-*value 0.9999 | z-value -2.167  *p-*value 0.9898 | z-value -1.943  *p-*value 0.9987 |
| week5 |  |  |  |  |  | z-value 0.475  *p-*value 1.0000 | z-value 0.546  *p-*value 1.0000 | z-value 1.168  *p-*value 1.0000 | z-value 1.934  *p-*value 0.9989 | z-value  -2.339  *p-*value 0.9656 | z-value  -1.031  *p-*value 1.0000 |
| week6 |  |  |  |  |  |  | z-value 0.055  *p-*value 1.0000 | z-value 0.896  *p-*value 1.0000 | z-value 1.715  *p-*value 0.9999 | z-value  -2.161  *p-*value 0.9903 | z-value  -0.686  *p-*value 1.0000 |
| week7 |  |  |  |  |  |  |  | z-value 0.889  *p-*value 1.0000 | z-value 1.742  *p-*value 0.9999 | z-value  -2.186  *p-*value 0.9882 | z-value  -0.678  *p-*value 1.0000 |
| week8 |  |  |  |  |  |  |  |  | z-value  0.539  *p-*value 1.0000 | z-value  -1.072  *p-*value 1.0000 | z-value  0.359  *p-*value 1.0000 |
| week9 |  |  |  |  |  |  |  |  |  | z-value  -0.627  *p-*value 1.0000 | z-value 1.049  *p-*value 1.0000 |
| week10 |  |  |  |  |  |  |  |  |  |  | z-value  -1.582  *p-*value 1.0000 |
| week11 |  |  |  |  |  |  |  |  |  |  |  |
| 22°C | |  |  |  |  |  |  |  |  |  |  |
| week1 |  | z-value 2.018  *p-*value 0.9973 | z-value 1.613  *p-*value 1.0000 | z-value 2.589  *p-*value 0.8746 | z-value  2.575  *p-*value 0.8819 | z-value 3.777  *p-*value 0.0849 | z-value 4.502  *p-*value 0.0052 | z-value 0.001  *p-*value 1.0000 | z-value 3.644  *p-*value 0.1285 | z-value 4.188  *p-*value 0.0193 | z-value 3.941  *p-*value 0.0485 |
| week2 |  |  | z-value -0.414 1.0000 | z-value 0.226 1.0000 | z-value 1.496 1.0000 | z-value 2.267 0.9785 | z-value 2.622 0.8565 | z-value 0.001 1.0000 | z-value 2.961 0.6004 | z-value -2.744 0.7767 | z-value -2.678 0.8220 |
| week3 |  |  |  | z-value 0.688  *p-*value 1.0000 | z-value 1.743  *p-*value 0.9999 | z-value  2.599  *p-*value 0.8692 | z-value 3.023  *p-*value 0.5464 | z-value 0.001  *p-*value 1.0000 | z-value 3.119  *p-*value 0.4626 | z-value  -3.062  *p-*value 0.5120 | z-value  -2.956  *p-*value 0.6045 |
| week4 |  |  |  |  | z-value 1.413  *p-*value 1.0000 | z-value 2.210  *p-*value 0.9858 | z-value 2.593  *p-*value 0.8726 | z-value 0.001  *p-*value 1.0000 | z-value 2.917  *p-*value 0.6381 | z-value -2.706  *p-*value 0.8037 | z-value  -2.635  *p-*value 0.8488 |
| week5 |  |  |  |  |  | z-value 0.264  *p-*value 1.0000 | z-value 0.288  *p-*value 1.0000 | z-value 0.001  *p-*value 1.0000 | z-value 1.772  *p-*value 0.9998 | z-value  -0.683  *p-*value 1.0000 | z-value  -0.638  *p-*value 1.0000 |
| week6 |  |  |  |  |  |  | z-value 0.009  *p-*value 1.0000 | z-value 0.001  *p-*value 1.0000 | z-value 1.706  *p-*value 0.9999 | z-value  -0.499  *p-*value 1.0000 | z-value  -0.638  *p-*value 1.0000 |
| week7 |  |  |  |  |  |  |  | z-value  0.001  *p-*value 1.0000 | z-value 1.752  *p-*value 0.9999 | z-value  -0.535  *p-*value 1.0000 | z-value  -0.678  *p-*value 1.0000 |
| week8 |  |  |  |  |  |  |  |  | z-value  0.001  *p-*value 1.0000 | z-value 0.001  *p-*value 1.0000 | z-value 0.001  *p-*value 1.0000 |
| week9 |  |  |  |  |  |  |  |  |  | z-value 1.387  *p-*value 1.0000 | z-value 1.238  *p-*value 1.0000 |
| week10 |  |  |  |  |  |  |  |  |  |  | z-value 0.170  *p-*value 1.0000 |
| week11 |  |  |  |  |  |  |  |  |  |  |  |
| 24°C | |  |  |  |  |  |  |  |  |  |  |
| week1 |  | z-value -1.171  *p-*value 1.0000 | z-value 0.222  *p-*value 1.0000 | z-value 1.932  *p-*value 0.9989 | z-value 3.110  *p-*value 0.4709 | z-value 4.426  *p-*value 0.0072 | z-value 6.933  *p-*value <0.0001 | z-value 3.094  *p-*value 0.4845 | z-value 6.238  *p-*value <0.0001 | z-value 5.579  *p-*value <0.0001 | z-value 5.926  *p-*value <0.0001 |
| week2 |  |  | z-value 1.257  *p-*value 1.0000 | z-value 2.913  *p-*value 0.6417 | z-value 3.659  *p-*value 0.1228 | z-value 5.071  *p-*value 0.0003 | z-value 7.404  *p-*value <0.0001 | z-value 3.567  *p-*value 0.1610 | z-value 6.599  *p-*value <0.0001 | z-value  -6.022  *p-*value <0.0001 | z-value  -6.405  *p-*value <0.0001 |
| week3 |  |  |  | z-value 1.529  *p-*value 1.0000 | z-value 2.890  *p-*value 0.6609 | z-value 4.051  *p-*value 0.0325 | z-value 6.616  *p-*value <0.0001 | z-value 2.925  *p-*value 0.6318 | z-value 6.066  *p-*value <0.0001 | z-value  -5.354  *p-*value 0.0001 | z-value  -5.649  *p-*value <0.0001 |
| week4 |  |  |  |  | z-value 2.034  *p-*value 0.9968 | z-value 3.003  *p-*value 0.5632 | z-value 5.831  *p-*value <0.0001 | z-value 2.183  *p-*value 0.9884 | z-value 5.495  *p-*value <0.0001 | z-value  -4.633 *p-*value 0.0029 | z-value  -4.870  *p-*value 0.0009 |
| week5 |  |  |  |  |  | z-value 0.301  *p-*value 1.0000 | z-value 3.032  *p-*value 0.5382 | z-value 0.360  *p-*value 1.0000 | z-value 3.586  *p-*value 0.1524 | z-value  -2.357  *p-*value 0.9615 | z-value  -2.332  *p-*value 0.9671 |
| week6 |  |  |  |  |  |  | z-value 3.122  *p-*value 0.4607 | z-value 0.131  *p-*value 1.0000 | z-value 2.309  *p-*value 0.9716 | z-value  -2.339  *p-*value 0.9656 | z-value  -2.329  *p-*value 0.9676 |
| week7 |  |  |  |  |  |  |  | z-value  -2.405  *p-*value 0.9492 | z-value 1.182  *p-*value 1.0000 | z-value 0.451  *p-*value 1.0000 | z-value 0.648  *p-*value 1.0000 |
| week8 |  |  |  |  |  |  |  |  | z-value 3.079  *p-*value 0.4973 | z-value  -1.846  *p-*value 0.9996 | z-value  -1.775  *p-*value 0.9998 |
| week9 |  |  |  |  |  |  |  |  |  | z-value 1.495  *p-*value 1.0000 | z-value 1.692  *p-*value 0.9999 |
| week10 |  |  |  |  |  |  |  |  |  |  | z-value  -0.158  *p-*value 1.0000 |
| week11 |  |  |  |  |  |  |  |  |  |  |  |
| 28°C | |  |  |  |  |  |  |  |  |  |  |
| week1 |  | z-value 3.077  *p-*value 0.4989 | z-value 0.193  *p-*value 1.0000 | z-value  3.033  *p-*value  0.0001 | z-value 2.965  *p-*value 0.5968 | z-value 4.586  *p-*value 0.0036 | z-value 6.756  *p-*value <0.0001 | z-value 4.065  *p-*value 0.0309 | z-value 5.524  *p-*value <0.0001 | z-value 5.503  *p-*value <0.0001 | z-value 5.188  *p-*value 0.0002 |
| week2 |  |  | z-value -2.763  *p-*value 0.7631 | z-value 2.072  *p-*value 0.9955 | z-value 0.776  *p-*value 1.0000 | z-value 1.801  *p-*value 0.9998 | z-value 3.890  *p-*value 0.0579 | z-value 2.403  *p-*value 0.9497 | z-value 3.237  *p-*value 0.3665 | z-value  -3.404  *p-*value 0.2490 | z-value  -2.891  *p-*value 0.6607 |
| week3 |  |  |  | z-value 4.998  *p-*value 0.0005 | z-value 2.783  *p-*value 0.7481 | z-value 4.289  *p-*value 0.0128 | z-value 6.425  *p-*value <0.0001 | z-value 3.931  *p-*value 0.0502 | z-value 5.298  *p-*value 0.0001 | z-value  -5.300  *p-*value 0.0001 | z-value  -4.961  *p-*value 0.0006 |
| week4 |  |  |  |  | z-value  2.773  *p-*value  1.0000 | z-value -0.050  *p-*value 1.0000 | z-value 2.089  *p-*value 0.9947 | z-value 1.179  *p-*value 1.0000 | z-value 1.686  *p-*value 1.0000 | z-value  -1.953  *p-*value 0.9986 | z-value  -1.322  *p-*value 1.0000 |
| week5 |  |  |  |  |  | z-value 0.717  *p-*value 1.0000 | z-value 2.456  *p-*value 0.9329 | z-value  1.623  *p-*value 1.0000 | z-value 2.112  *p-*value 0.9935 | z-value -2.338  *p-*value 0.9658 | z-value  -1.803  *p-*value 0.9998 |
| week6 |  |  |  |  |  |  | z-value 1.969  *p-*value 0.9983 | z-value 1.164  *p-*value 1.0000 | z-value 1.622  *p-*value 1.0000 | z-value  -1.885  *p-*value 0.9993 | z-value  -1.279  *p-*value 1.0000 |
| week7 |  |  |  |  |  |  |  | z-value  -0.257  *p-*value 1.0000 | z-value -0.121  *p-*value 1.0000 | z-value -0.232  *p-*value 1.0000 | z-value 0.474  *p-*value 1.0000 |
| week8 |  |  |  |  |  |  |  |  | z-value 0.148  *p-*value 1.0000 | z-value  -0.417  *p-*value 1.0000 | z-value 0.122  *p-*value 1.0000 |
| week9 |  |  |  |  |  |  |  |  |  | z-value -0.319  *p-*value 1.0000 | z-value 0.320  *p-*value 1.0000 |
| week10 |  |  |  |  |  |  |  |  |  |  | z-value  -0.627  *p-*value 1.0000 |
| week11 |  |  |  |  |  |  |  |  |  |  |  |

**Table S5-** LMM analysis. Effects of Treatment on routine metabolic rates. Comparisons between treatments. Est-Estimates, Std error-Standard error.

| RMR | | | | |
| --- | --- | --- | --- | --- |
|  | chisq | p-value |  |  |
| Treatment | 4.0926 | 0.2516 |  |  |
|  | Est | Std. Error | z-value | p-value |
| 19°C-22°C | 80.26 | 60.76 | 1.321 | 0.549 |
| 19°C-24°C | 159.65 | 63.06 | 2.532 | 0.055 |
| 19°C-28°C | 69.65 | 66.13 | 1.053 | 0.718 |
| 22°C-24°C | 79.39 | 60.76 | 1.307 | 0.558 |
| 22°C-28°C | -10.6 | 63.95 | -0.166 | 0.998 |
| 24°C-28°C | -90 | 66.13 | -1.361 | 0.524 |

**Table S6-** GLMM and LMM analysis. Effects of Treatment on CS, LDH, CAT, GST and LPO. Comparisons between treatments. Est-Estimates, Std error-Standard error.

| CS muscle | | | | | |
| --- | --- | --- | --- | --- | --- |
|  | chisq | p-value |  | |  |
| Treatment | 10.383 | 0.0515 |  |  | |
| LDH muscle | | | | | |
|  | chisq | p-value |  |  | |
| Treatment | 3.2987 | 0.3478 |  |  | |
| CAT gills | | | | | |
|  | chisq | p-value |  |  | |
| Treatment | 1.4589 | 0.6918 |  |  | |
|  | Estimate | Std. Error | z-value | p-value | |
| 19°C-22°C | 0.1354 | 0.3666 | 0.369 | 0.983 | |
| 19°C-24°C | -0.2620 | 0.3360 | -0.780 | 0.863 | |
| 19°C-28°C | -0.1362 | 0.3542 | -0.384 | 0.981 | |
| 22°C-24°C | -0.3974 | 0.3491 | -1.138 | 0.665 | |
| 22°C-28°C | -0.2716 | 0.3666 | -0.741 | 0.880 | |
| 24°C-28°C | 0.1259 | 0.3360 | 0.375 | 0.982 | |
| GST muscle | | | | | |
|  | chisq | p-value |  |  | |
| Treatment | 0.3206 | 0.9561 |  |  | |
|  | Estimate | Std. Error | z-value | p-value | |
| 19°C-22°C | -0.01532 | 0.0930 | -0.165 | 0.9984 | |
| 19°C-24°C | -0.03574 | 0.0873 | -0.409 | 0.9769 | |
| 19°C-28°C | 0.00807 | 0.0824 | 0.098 | 0.9997 | |
| 22°C-24°C | -0.02043 | 0.0923 | -0.221 | 0.9962 | |
| 22°C-28°C | 0.02339 | 0.0876 | 0.267 | 0.9933 | |
| 24°C-28°C | 0.04382 | 0.0815 | 0.537 | 0.9499 | |
| GST brain | | | | | |
|  | chisq | p-value |  |  | |
| Treatment | 12.523 | 0.0058 |  |  | |
|  | Estimate | Std. Error | z-value | p-value | |
| 19°C-22°C | -0.35180 | 0.21943 | -1.603 | 0.3694 | |
| 19°C-24°C | -0.08412 | 0.17070 | -0.493 | 0.9596 | |
| 19°C-28°C | 0.26048 | 0.15264 | 1.706 | 0.3133 | |
| 22°C-24°C | 0.26768 | 0.21310 | 1.256 | 0.5839 | |
| 22°C-28°C | 0.61228 | 0.19893 | 3.078 | 0.0109 | |
| 24°C-28°C | 0.34459 | 0.14340 | 2.403 | 0.0739 | |
| GST gills | | | | | |
|  | chisq | p-value |  |  | |
| Treatment | 9.7456 | 0.0208 |  |  | |
|  | Estimate | Std. Error | z-value | p-value | |
| 19°C-22°C | 1.044 | 0.245 | 0.183 | 0.9978 | |
| 19°C-24°C | 0.847 | 0.176 | -0.798 | 0.8551 | |
| 19°C-28°C | 0.589 | 0.122 | -2.549 | 0.0527 | |
| 22°C-24°C | 0.812 | 0.177 | -0.959 | 0.7727 | |
| 22°C-28°C | 0.564 | 0.123 | -2.630 | 0.0424 | |
| 24°C-28°C | 0.695 | 0.131 | -1.929 | 0.2156 | |
| GST between tissues | | | | | |
|  | chisq | p-value |  |  | |
| tissue | 1266.5 | ˂0.001 |  |  | |
|  | Estimate | Std. Error | z-value | p-value | |
| Brain-Gills | 0.155 | 0.0171 | -16.835 | <.0001 | |
| Brain-Muscle | 9.147 | 1.0684 | 18.951 | <.0001 | |
| Gills-Muscle | 59.192 | 6.7877 | 35.586 | <.0001 | |
| LPO muscle | | | | | |
|  | chisq | p-value |  |  | |
| Treatment | 1.0948 | 0.7783 |  |  | |
|  | Estimate | Std. Error | z-value | p-value | |
| 19°C-22°C | -0.1276 | 0.4214 | -0.303 | 0.990 | |
| 19°C-24°C | 0.2757 | 0.4693 | 0.587 | 0.936 | |
| 19°C-28°C | 0.2344 | 0.4606 | 0.509 | 0.957 | |
| 22°C-24°C | 0.4033 | 0.4554 | 0.886 | 0.812 | |
| 22°C-28°C | 0.3620 | 0.4464 | 0.811 | 0.849 | |
| 24°C-28°C | -0.0413 | 0.4918 | -0.084 | 1.000 | |
| LPO gills | | | | | |
|  | chisq | p-value |  |  | |
| Treatment | 8.7074 | 0.03344 |  |  | |
|  | Estimate | Std. Error | z-value | p-value | |
| 19°C-22°C | 0.72221 | 0.41611 | 1.736 | 0.3029 | |
| 19°C-24°C | 0.08939 | 0.34278 | 0.261 | 0.9937 | |
| 19°C-28°C | -0.46464 | 0.34278 | -1.356 | 0.5250 | |
| 22°C-24°C | -0.63282 | 0.40862 | -1.549 | 0.4059 | |
| 22°C-28°C | -1.18685 | 0.40862 | -2.905 | 0.0188 | |
| 24°C-28°C | -0.55404 | 0.33363 | -1.661 | 0.3424 | |
| LPO between tissues | | | | | |
|  | chisq | p-value |  |  | |
| tissue | 18.611 | ˂0.001 |  |  | |

**Table S7**- Behavioral categories analyzed for *Sparus aurata*.

| Behavioral categories | Behavior description |
| --- | --- |
| Chase (frequency) | A fish attacks or pursues a feeing fish. |
| Bite (frequency) | Individual bites conspecific, after chasing. |
| Swimming (time, seconds) | Active swimming- movement against the current in which the individual uses the pectoral and caudal fins to move.  Surface touching- the individual swims and touches the surface of the water. |
| Inside the shelter (seconds) | Individual remains motionless or swimming inside the shelter |
